# The mitochondrial RNA polymerase POLRMT promotes skin squamous cell carcinoma cell growth

**DOI:** 10.1038/s41420-022-01148-5

**Published:** 2022-08-03

**Authors:** Yulong Wang, Li Ou, Xirong Li, Tingyu Zheng, Wei-pei Zhu, Ping Li, Lijun Wu, Tianlan Zhao

**Affiliations:** 1 Department of Plastic and Cosmetic Surgery, The Second Affiliated Hospital of Soochow University, Suzhou, China; 2 Department of Burn and Plastic Surgery, Jining First People’s Hospital, Jining, China; 3 Department of Gynecology and Obstetrics, The Second Affiliated Hospital of Soochow University, Suzhou, China; 4 Department of Radiotherapy and Oncology, Affiliated Kunshan Hospital of Jiangsu University, Kunshan, China

**Keywords:** Squamous cell carcinoma, Targeted therapies

## Abstract

RNA polymerase mitochondrial (POLRMT) expression and the potential biological functions in skin squamous cell carcinoma (SCC) were explored. We showed that POLRMT is significantly elevated in skin SCC. Genetic depletion of POLRMT, using shRNA-induced knockdown or CRISPR/Cas9-mediated knockout (KO), resulted in profound anti-skin SCC cell activity. In patient-derived primary skin SCC cells or immortalized lines (A431 and SCC-9), POLRMT shRNA or KO potently suppressed mitochondrial DNA (mtDNA) transcription and suppressed cell viability, proliferation and migration. POLRMT shRNA or KO impaired mitochondrial functions in different skin SCC cells, leading to production of ROS (reactive oxygen species), depolarization of mitochondria and depletion of ATP. Moreover, mitochondrial apoptosis cascade was induced in POLRMT-depleted skin SCC cells. IMT1, a POLRMT inhibitor, largely inhibited proliferation and migration, while inducing depolarization of mitochondria and apoptosis in primary skin SCC cells. Contrarily, ectopic overexpression of POLRMT increased mtDNA transcription and augmented skin SCC cell growth. Importantly, POLRMT shRNA adeno-associated virus injection robustly hindered growth of the subcutaneous A431 xenografts in mice. In the POLRMT shRNA virus-treated A431 xenograft tissues, POLRMT depletion, mtDNA transcription inhibition, cell apoptosis, lipid peroxidation and ATP depletion were detected. Together, overexpressed POLRMT increases mtDNA transcription and promotes skin SCC growth.

## Introduction

Skin squamous cell carcinoma (SCC) is characterized by abnormal, rapid growth of squamous cells [1, 2]. The present clinical therapies for skin SCC include surgery (tumor resection), chemotherapy and radiotherapy [3–5]. For the advanced SCC patients, however the prognosis is often extremely poor [3–5]. Immunotherapy and molecule-targeted therapies are being tested for skin SCC patients [3–5].

Mitochondria are the hub for oxidative phosphorylation (OXPHOS), providing energy for cells to survive and growth [6–8]. Mitochondrion has its own genome mitochondrial DNA (mtDNA) [8–11]. It encodes several key components of mitochondrial electron transport chain of OXPHOS system [8–11]. Abnormally enhanced mtDNA transcription and OXPHOS system are essential for meeting the enhanced energy demand of rapidly growing cancer cells [8, 12, 13]. Increased mtDNA transcription and OXPHOS are commonly observed in skin SCC and are closely related to the occurrence and development of cancer [14–16]. Targeting mtDNA transcription and OXPHOS could efficiently inhibit human cancer cell growth [6, 8, 9, 17].

RNA polymerase mitochondrial (POLRMT) is vital for mtDNA transcription [18, 19]. POLRMT is also key for the integrity of mitochondrial transcription machinery [18, 19]. In addition POLRMT can synthesize RNA primers required for mtDNA replication [20, 21]. Recent studies have indicated POLRMT as a promising therapeutic oncotarget [9, 22–24]. Overexpressed POLRMT increased mtDNA transcription and mitochondrial OXPHOS, promoting cancer cell growth [9, 22–24]. The current study will support that overexpressed POLRMT is required for growth of the skin SCC cells.

## Results

### POLRMT expression is elevated in skin SCC

**Fig. 1:**
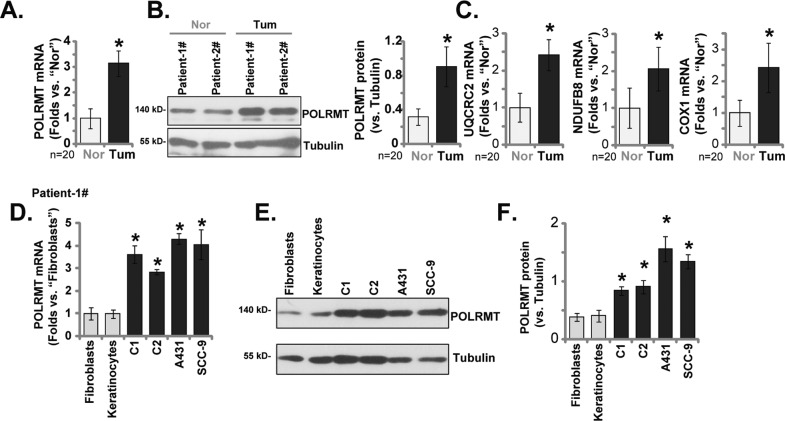
POLRMT expression is elevated in skin SCC. *POLRMT* mRNA and protein expression as well as *NDUFB8*, *UQCRC2* and *COXI* mRNA expression in skin SCC tumor tissues (“Tum”) and matched adjacent normal skin tissues (“Nor”) from a set of twenty (*n* = 20) primary skin SCC patients was tested by qRT-PCR and Western blotting assays (**A**–**C**). *POLRMT* mRNA and protein expression in the listed skin SCC cells, primary human skin fibroblasts (“Fibroblasts”) and keratinocytes (“Keratinocytes”) was tested (**D**–**F**). Data were mean ± standard deviation (SD). *P < 0.05 versus “Nor” tissues/“Fibroblasts”.

First, the qRT-PCR results showed that the *POLRMT* mRNA expression in twenty skin SCC tumor tissues was about three folds of that in the normal skin tissues (Fig. 1A). Moreover, POLRMT protein is upregulated in skin SCC tissues of the two representative SCC patients (Patient-1# to Patient-2#, Fig. 1B). Quantification results of all twenty patients’ POLRMT blotting data further confirmed that POLRMT protein is significantly elevated in the tumor tissues (Fig. 1B). We found that the mRNA expression of POLRMT-dependent mitochondrial transcripts, including *NDUFB8*, *UQCRC2* and *COXI* [9, 22], was also significantly elevated in the skin SCC tumor tissues (Fig. 1C).

POLRMT expression in skin SCC cells and normal skin cells was examined. In A431 cells and SCC-9 cells as well as in primary skin SCC cells [derived from Patient-1# (“C1” cells) and Patient-2# (“C2” cells), the mRNA and protein expression of POLRMT was robustly higher than it in the primary human skin keratinocytes and fibroblasts (Fig. 1D–F)]. These results together confirmed elevated POLRMT expression in skin SCC.

### POLRMT silencing or KO inhibits primary skin SCC cell proliferation and migration

**Fig. 2:**
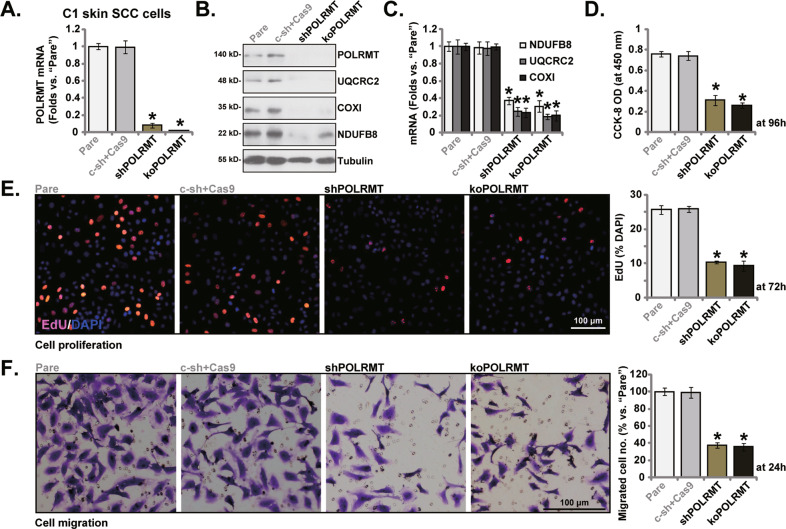
POLRMT silencing or KO inhibits skin SCC cell proliferation and migration. Stable C1 primary human skin SCC cells, expressing the lentiviral POLRMT shRNA (“shPOLRMT”), the CRISPR/Cas9-POLRMT-KO construct (“koPOLRMT”) or scramble control shRNA plus Cas9 control construct (“c-sh+Cas9”), were established; Expression of listed genes and proteins was tested (**A**–**C**); Cells were further cultured for the designated time periods, cell viability, proliferation and migration were tested by CCK-8 (**D**), nuclear EdU staining (**E**) and “Transwell” (**F**) assays, respectively. “Pare” stands for parental control C1 primary cells. *P < 0.05 versus “Pare” cells. Scale bar = 100 μm (**E**, **F**).

To silence POLRMT in skin SCC cells, the POLRMT shRNA-encoding lentiviral particles (from Dr. Shi [22]) were transduced to C1 primary human skin SCC cells, and stable cells (“shPOLRMT”) were thereafter formed with puromycin selection. Alternatively, to Cas9-expressing C1 primary skin SCC cells the CRISPR/Cas9-POLRMT-KO construct (from Dr. Shi [22]) was transduced, and stable cells (“koPOLRMT”) were established. qRT-PCR assays, Fig. 2A, showed that *POLRMT* mRNA expression decreased over 90–95% in shPOLRMT and koPOLRMT C1 skin SCC cells, where POLRMT protein depletion was detected as well (Fig. 2B). Expression of POLRMT-dependent mitochondrial transcripts, including *NDUFB8*, *UQCRC2* and *COXI* [9, 22], was also dramatically decreased in POLRMT-shRNA/-KO C1 primary cells (Fig. 2B and C).

Next, we studied whether POLRMT depletion could alter skin SCC cell functions. CCK-8 assays showed that the viability (CCK-8 OD) was dramatically reduced in shPOLRMT and koPOLRMT C1 cells (Fig. 2D). Moreover, POLRMT shRNA or KO robustly decreased the percentage of EdU positively-stained nuclei, suggesting that POLRMT depletion inhibited C1 cell proliferation (Fig. 2E). Furthermore, “Transwell” assay results demonstrated that POLRMT shRNA or KO largely suppressed C1 cell in vitro migration (Fig. 2F). Scramble control shRNA plus Cas9 construct (“c-sh+Cas9”) control treatment failed to affect POLRM and mitochondrial transcripts expression (Fig. 2A–C) as well as C1 cell functions (Fig. 2D–F).

### POLRMT silencing or KO disrupts mitochondrial functions and provokes apoptosis in primary skin SCC cells

**Fig. 3:**
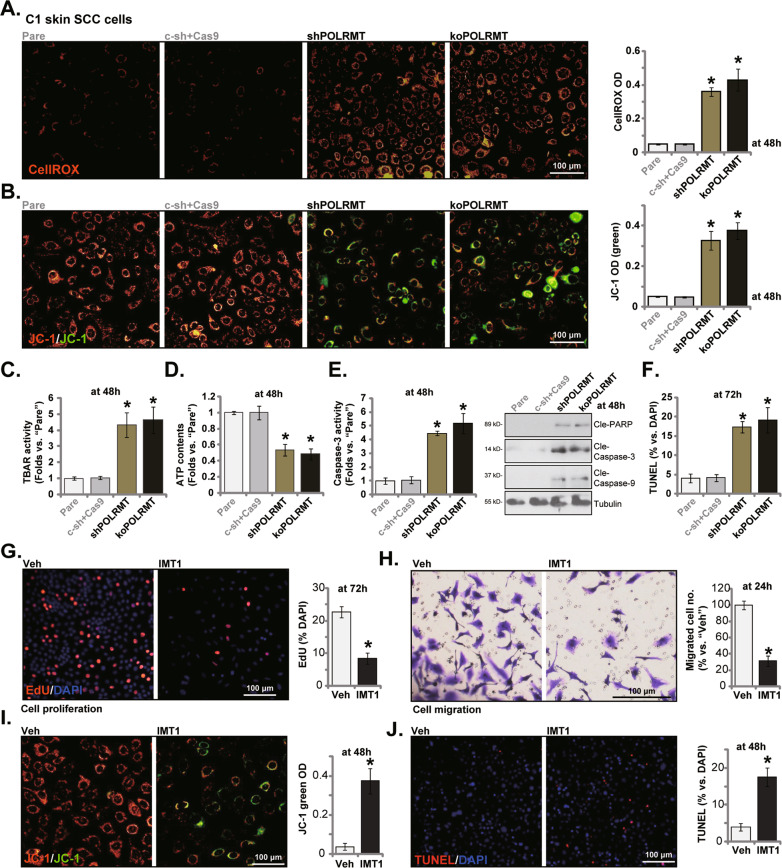
POLRMT silencing or KO disrupts mitochondrial functions and provokes apoptosis in primary skin SCC cells. Stable C1 primary human skin SCC cells, expressing the lentiviral POLRMT shRNA (“shPOLRMT”), the CRISPR/Cas9-POLRMT-KO construct (“koPOLRMT”) or scramble control shRNA plus Cas9 control construct (“c-sh+Cas9”) were established and cultivated for the designated time periods; The cellular ROS contents were analyzed by measuring CellROX fluorescence intensity (**A**), Mitochondrial depolarization and lipid peroxidation were examined by JC-1 dye (**B**) and TBAR activity (**C**) assays, respectively; The cellular ATP contents were measured as well (**D**). Caspase-apoptosis activation was tested by the listed assays (**E**, **F**). The C1 primary skin SCC cells were treated with the POLRMT inhibitor IMT1 (500 nM) or the vehicle control (0.1% DMSO, “Veh”) for applied time periods, cell proliferation, migration, depolarization of mitochondria and apoptosis were measured by nuclear EdU/DAPI staining (**G**), “Transwell” (**H**), JC-1 staining (**I**) and TUNEL/DAPI staining (**J**) assays, respectively. “Pare” stands for parental control C1 primary cells. *P < 0.05 versus “Pare” cells/“Veh” treatment. Scale bar = 100 μm.

Considering that POLRMT is essential for mtDNA transcription and mitochondrial integrity [18–21], we examined whether POLRMT depletion affected mitochondrial functions in skin SCC cells. As shown, POLRMT shRNA or KO (see Fig. 2) led to dramatic ROS production in C1 primary skin SCC cells, elevating CellROX red fluorescence intensity [25] (Fig. 3A). Moreover, the accumulation of green JC-1 monomers indicated depolarization of mitochondria in shPOLRMT and koPOLRMT C1 primary cells (Fig. 3B). In addition, TBAR intensity was increased in POLRMT-depleted cells, supporting lipid peroxidation (Fig. 3C). The cellular ATP contents were decreased as well following POLRMT silencing/KO (Fig. 3D). Thus POLRMT depletion disrupted mitochondrial functions in skin SCC cells.

In human cancer cells mitochondrial dysfunction will lead to mitochondrial apoptosis cascade induction [26–29]. We found that the caspase-3 activity was boosted in shPOLRMT and koPOLRMT C1 primary cells (Fig. 3E). The percentage of TUNEL-positive nuclei e was increased in shPOLRMT and koPOLRMT C1 SCC cells (Fig. 3F), supporting significant apoptosis activation. As expected, c-sh+Cas9 control treatment (see Fig. 2) failed to disrupt mitochondrial functions (Fig. 3A–D) and provoke apoptosis in C1 cells (Fig. 3E and F).

To further support our hypothesis, we showed that IMT1, a first-in-class specific and noncompetitive POLRMT inhibitor [9, 30], robustly suppressed proliferation (Fig. 3G) and migration (Fig. 3H) in C1 cells. Moreover, the POLRMT inhibitor caused depolarization of mitochondria and green JC-1 monomers increasing (Fig. 3I). Apoptosis induction, evidenced by the increased TUNEL nuclei (Fig. 3J), was observed in IMT1-treated C1 SCC cells. These results further supported an important function of POLRMT in skin SCC cells.

### POLRMT shRNA exerts anti-cancer activity in other skin SCC cells

**Fig. 4:**
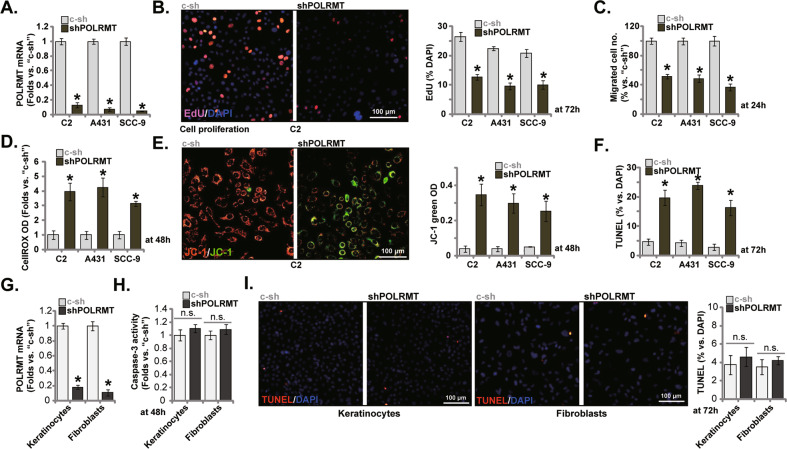
POLRMT shRNA exerts anti-cancer activity in other skin SCC cells. The listed skin SCC cells, primary human skin fibroblasts (“Fibroblasts”) and keratinocytes (“Keratinocytes”), stably expressing the lentiviral POLRMT shRNA (“shPOLRMT”) or the scramble control shRNA (“sh-C”), were established, *POLRMT* mRNA expression was tested (**A** and **G**). Cells were further cultivated for applied time periods, cell proliferation, migration, ROS production, depolarization of mitochondria and apoptosis were tested by nuclear EdU staining (**B**), “Transwell” (**C**), CellROX staining (**D**), JC-1 staining (**E**) and caspase-3 activity (**H**)-TUNEL staining (**F** and **I**) assays, respectively, and results were quantified. *P < 0.05 versus “sh-C” cells. Scale bar = 100 μm (**B**, **E**, and **I**).

Next, we examined whether POLRMT depletion could affect functions of other skin SCC cells. In primary skin SCC cells derived from another patient, C2, and in the established lines (A431 and SCC-9 [31]), POLRMT shRNA lentiviral particles were added. Puromycin was then included to select stable cells, namely “shPOLRMT” cells. As shown *POLRMT* mRNA was silenced in the shPOLRMT cells (Fig. 4A). In the SCC cells, EdU staining and “Transwell” assay results showed that POLRMT shRNA remarkably suppressed cell proliferation (Fig. 4B) and migration (Fig. 4C). Moreover, POLRMT shRNA induced ROS accumulation (CellROX increasing, Fig. 4D), mitochondrial membrane potential loss (Fig. 4E) and apoptosis (TUNEL-nuclei increasing, Fig. 4F) in C2 primary cells and immortalized cell lines. Therefore, POLRMT silencing induced profound anti-cancer activity in skin SCC cells.

The POLRMT shRNA lentiviral particles were added to skin keratinocytes and fibroblasts, resulting in significant *POLRMT* mRNA silencing (“shPOLRMT”) after selection (Fig. 4G). As shown, shPOLRMT treatment was unable to increase the caspase-3 activity (Fig. 4H) and TUNE-nuclei percentage (Fig. 4I). Therefore, POLRMT silencing failed to provoke apoptosis in non-cancerous skin cells, supporting a cancer cell specific effect.

### POLRMT overexpression exerts pro-cancer activity in primary skin SCC cells

**Fig. 5:**
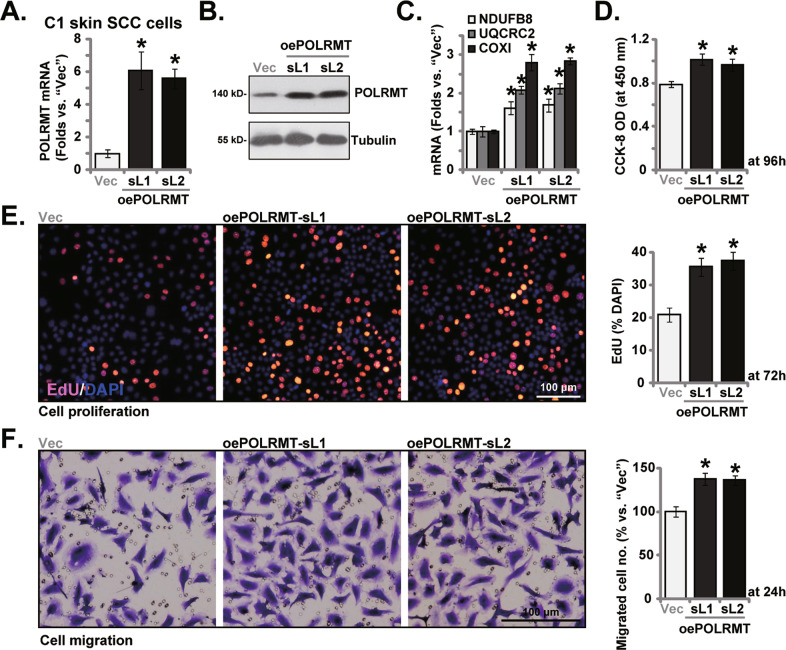
POLRMT overexpression exerts pro-cancer activity in primary skin SCC cells. C1 primary skin SCC cells expressing the lentiviral POLRMT-expressing construct (oePOLRMT-sL1 and oePOLRMT-sL2, representing two stable selections) or the empty vector (“Vec”) were established, expression of listed genes and proteins was shown (**A**–**C**); Cells were further cultivated for applied time periods, cell viability, proliferation and migration were tested by CCK-8 (**D**), nuclear EdU staining (**E**) and “Transwell” (**F**) assays, respectively. *P < 0.05 versus “Vec” cells. Scale bar = 100 μm (**E**, **F**).

Next we exogenously increased POLRMT expression by transducing a lentiviral POLRMT-overexpressing construct (from Dr. Shi [22]) to C1 primary skin SCC cells. Stable cells were formed with selection using puromycin: oePOLRMT-sL1 and oePOLRMT-sL2 (representing two stable selections). Figure 5A showed that *POLRMT* mRNA was dramatically elevated in the oePOLRMT C1 skin SCC cells. Figure 5B results further confirmed POLRMT protein overexpression. POLRMT-dependent mitochondrial transcripts, including *NDUFB8*, *COXI* and *UQCRC2*, were increased in POLRMT-overexpressed C1 cells (Fig. 5C). POLRMT overexpression increased C1 cell viability (Fig. 5D) and proliferation (Fig. 5E). Moreover, cell migration was augmented in oePOLRMT C1 cells (Fig. 5F). Therefore, ectopic overexpression of POLRMT exerted pro-cancer activity in primary skin SCC cells.

### POLRMT shRNA virus injection hinders subcutaneous A431 xenograft growth

**Fig. 6:**
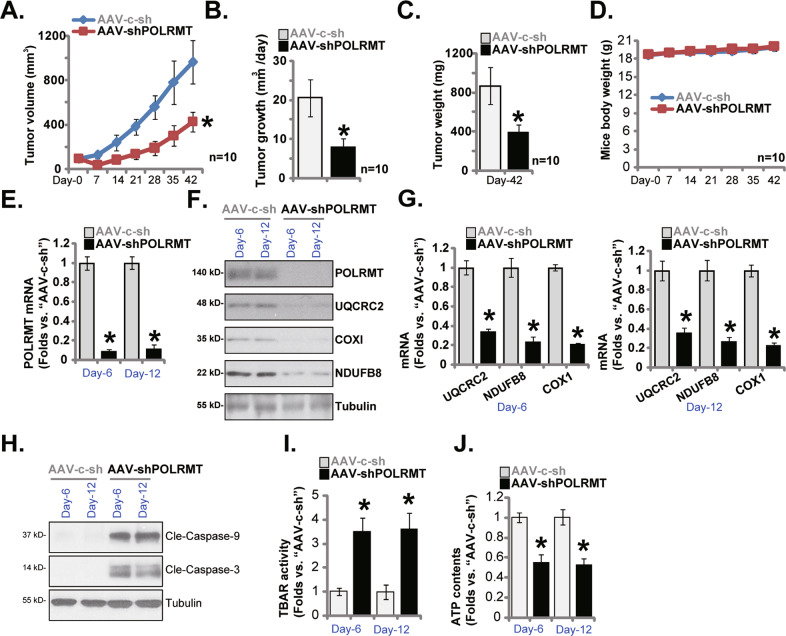
POLRMT shRNA virus injection hinders subcutaneous A431 xenograft growth. The nude mice bearing the subcutaneous A431 xenografts were subject to intratumoral injection of the POLRMT-shRNA-expressing AAV (“AAV-shPOLRMT”) or scramble control shRNA AAV (“AAV-c-sh”). Virus was injected daily for four consecutive days. Tumor volumes (**A**) and mice body weights (**D**) were recorded weekly for six consecutive weeks (“Day-0” to “Day-42”). The estimated daily tumor growth, in mm^3^ per day, was calculated as described (**B**). All experimental animals were sacrificed by decapitation at “Day-42”, and xenograft tumors were carefully isolated and weighed individually (**C**). Expression of listed genes and proteins in the tumor lysates of the described tumors was tested by qRT-PCR (**E** and **G**) and Western blotting (**F** and **H**) assays. TBAR activity (**I**) and ATP contents (**J**) were tested as well. Data were mean ± standard deviation (SD). *P < 0.05 versus “AAV-c-sh” group.

A431 cells, at 6 × 10 ^6^ cells of each mouse, were injected *s.c*. to nude mice’s flanks. Subcutaneous A431 xenograft tumors were allowed to grow for 3 weeks and the volume of every xenograft was thereafter close to 0.1 cm^3^ (labeled as “Day-0”). To silence POLRMT expression in vivo, POLRMT-shRNA-expressing AAV (from Dr. Shi [22]) were intratumorally injected to subcutaneous A431 xenografts (“AAV-shPOLRMT” group). Control A431 xenografts were injected with the AAV expressing the scramble control shRNA (“AAV-c-sh” group, also from Dr. Shi [22]). Virus injection was carried out daily (“Day-0” to “Day-3”). When measuring tumor volumes weekly, we showed that AAV-shPOLRMT injection robustly inhibited subcutaneous A431 xenograft tumor growth (Fig. 6A). The volumes of AAV-shPOLRMT-injected A431 xenografts were significantly lower than those with injection of AAV-c-sh (Fig. 6A). Estimating daily tumor growth [22], Fig. 6B, found that AAV-shPOLRMT injection inhibited subcutaneous A431 xenograft growth. At Day-42, all experimental mice were killed by decapitation. A431 tumors were carefully removed and individually weighted. AAV-shPOLRMT A431 xenografts were remarkably lighter than AAV-c-sh tumors (Fig. 6C). Among the two groups there was no significant difference in the body weights (Fig. 6D).

On “Day-6” and “Day-12”, one mouse in each group was decapitated, and A431 tumors were carefully isolated. *POLRMT* mRNA (Fig. 6E) and protein (Fig. 6F) levels were dramatically decreased in AAV-shPOLRMT-injected A431 tumors. mRNA and protein levels of POLRMT-dependent mitochondrial transcripts, *NDUFB8*, *UQCRC2* and *COXI*, were also decreased (Fig. 6F and G). Conversely, cleaved-caspase-3/−9 levels were dramatically increased (Fig. 6H), supporting apoptosis induction. In AAV-shPOLRMT-injected A431 tumor tissues TBAR activity (lipid peroxidation intensity) was significantly augmented (Fig. 6I) and ATP contents (Fig. 6J) were decreased.

## Discussion

About 4% of skin SCC will metastasize, and these patients have a poor prognosis with 5-year survival rate <25% [1, 2, 32]. The novel and more efficient targeted therapies are urgently needed especially for skin SCC patients with advanced, metastatic and recurrent tumors [3–5]. Clinical studies have shown that immune CD1 inhibitors and epidermal growth factor receptor (EGFR) blockers, among others, displayed promising efficiency for skin SCC patients [33–35].

Recent studies have supported POLRMT as a potential oncogenic gene and therapeutic target of human cancer. Several inhibitors of mitochondrial transcription (IMTs) targeting POLRMT were developed [9]. IMTs impaired mtDNA transcription and inhibited oxidative phosphorylation, suppressing growth of different cancer xenografts [9]. Zhou et al., reported that POLRMT is overexpressed in non-small cell lung cancer [22]. Genetic silencing or depletion of POLRMT resulted in robust anti-NSCLC activity. Han et al., have recently shown that POLRMT overexpression in osteosarcoma is vital for cancer cell growth, representing as a novel molecular target [23].

POLRMT is a promising and novel therapeutic target of skin SCC. POLRMT expression is remarkably elevated in skin SCC tissues and cells. While in normal skin tissues and non-cancerous skin cells its expression is relatively low. Genetic depletion of POLRMT, using shRNA-induced knockdown or CRISPR/Cas9 method, resulted in robust anti-skin SCC cell activity. POLRMT shRNA or KO potently suppressed mtDNA transcription and inhibited skin SCC cell viability and proliferation. IMT1, a POLRMT inhibitor, largely inhibited skin SCC cell proliferation and migration, while inducing depolarization of mitochondria and apoptosis in primary skin SCC cells. Contrarily, further increasing POLRMT expression by a lentiviral construct enhanced mtDNA transcription and augmented skin SCC cell growth. Importantly, injection of AAV-packed POLRMT shRNA robustly inhibited subcutaneous A431 xenograft growth.

Like all other tumor cells, rapidly growing skin SCC cells require elevated mitochondrial function to provide energy. Lee et al., reported aberrant skin cancer cell proliferation was possibly due to enhanced mitochondrial biogenesis [36]. The mtDNA copy number as well as mitochondrial biogenesis–associated genes are elevated in skin cancers [36]. Dynamin-related protein 1 (Drp1) is upregulated in skin SCC. Silencing Drp1 inhibited skin SCC cell growth and led to G2 cell cycle arrest. Moreover, Drp1 silencing resulted in an elongated, hyper-fused mitochondrial network in skin SCC cells [37].

Here we show that overexpression POLRMT is vital for mitochondrial functions in skin SCC cells. Conversely, POLRMT shRNA or KO not only hindered mtDNA transcription, but also disrupted mitochondrial functions, causing significant ROS production, lipid peroxidation, depolarization of mitochondria and ATP depletion in skin SCC cells. Importantly, mitochondrial apoptosis cascade was induced in POLRMT-depleted skin SCC cells. In POLRMT–silenced A431 xenograft tissues, decreased mtDNA transcription, apoptosis induction, lipid peroxidation and ATP depletion were observed. Thus, overexpressed POLRMT promotes mtDNA transcription and increases mitochondrial functions, thereby accelerating skin SCC cell growth.

## Conclusion

Overexpressed POLRMT increases mtDNA transcription and promotes skin SCC cell growth.

## Materials and methods

### Chemicals and reagents

Puromycin and cell culture reagents were provided by Sigma (St. Louis, Mo). Cell counting kit -8 (CCK-8), EdU, DAPI, CellROX (Deep Red), TUNEL and JC-1 dyes were reported previously [23]. Antibodies for NDUFB8 (NADH:Ubiquinone Oxidoreductase Subunit B8, ab110242), UQCRC2 (Ubiquinol-Cytochrome C Reductase Core Protein 2, ab203832a) and POLRMT (ab228576) were provided by Abcam (Cambridge, MA). The antibody of cytochrome c oxidase subunit 1 (COXI, #55159) was provided by Cell Signaling Tech (Shanghai, China). Other antibodies were described elsewhere [22].

### Cell culture

Patient-derived primary skin SCC cells from two written-informed consent patients, “C1” and “C2”, the immortalized cell lines (A431 and SCC9), the primary human skin fibroblasts and human skin keratinocytes were provided from Dr. Liu [31]. The detailed protocols for cell culture were reported early [31, 38, 39]. The protocols were approved by the Ethics Committee of The Second Affiliated Hospital of Soochow University, in according to the principles of Declaration of Helsinki.

### Human tissues

Skin SCC tumor tissues along with the matched adjacent normal skin tissues were from a total of twenty (20) primary skin SCC patients. All patients underwent tumor resection surgeries at authors’ institution and each provided written-informed consent.

### Silencing or overexpression of POLRMT

The lentiviral particles encoding the POLRMT shRNA or the POLRMT-expressing construct were provided by Dr. Shi [22]. Skin SCC cells were maintained at 50% confluence and infected with the lentiviral particles. Puromycin was included for six passages. POLRMT expression was always tested.

### POLRMT knockout (KO)

The primary human SCC cells were transduced with a Cas-9-expressing construct (from Dr. Shi [22]) and stable cells were formed. Cells were further transfected with a CRISPR/Cas9-POLRMT-KO construct (provided by Dr. Shi [22]). Single stable POLRMT KO skin SCC cells were formed using the described protocol [22].

### Fluorescence dye assays

In brief, according to the manufacture’s protocols, skin SCC cells were cultivated at 50-60% confluence, stained with the designated fluorescence dyes (JC-1, CellROX, TUNEL, EdU and DAPI) and cultured for applied time periods. Fluorescence images were captured under a fluorescence microscopy (Leica).

**Other functional assays**, including the caspase-3 activity assay, Western blotting assay, mRNA detection by qRT-PCR were described in early studies [22, 31]. Measuring ATP contents was described previously [40]. The intensity of lipid peroxidation in cell and tissue lysates was examined through a thiobarbituric acid reactive substance (TBAR) kit (Cayman Chemical, MI). The reagent colorimetrically (at 540 nm) quantified lipid peroxidation and the formation malondialdehyde (MDA) [41]. The uncropped blotting images were presented in Fig. S1.

### Xenograft studies

As described [22, 38, 39], A431 skin SCC cells, at 6 × 10 ^6^ cells of each mouse [22], were injected subcutaneously (*s.c*.) to nude mice’s flanks (half male hale female, 5- to 6-week old, 18.4–19.1 g). All mice were provided and kept in the Laboratory Animal Center of Soochow University. Three weeks after cell implantation, A431 xenograft tumors were established and each tumor’s volume was close to 100 mm^3^. Afterwards, A431 xenograft mice were assigned into two random groups, intratumorally injected with POLRMT shRNA adeno-associated virus (AAV) or scramble control shRNA AAV (“AAV-c-sh”) (from Dr. Shi [22]). Virus was daily injected for 4 days. Tumor dimensions were measured by the described formula [23]. The protocols of handling experimental mice were according to institutional animal care and use committee (IACUC) and the Ethic Committee of Soochow University.

### Statistical analyses

The in vitro experiments were repeated five times and similar results were obtained each time, and in vitro data were mean ± standard deviation (SD, *n* = 5). Statistical analyses were described previously [22].

## Supplementary information

Authorship change_form

Figure S1

## Data Availability

All data are available upon request.
