# Supplementary figures and images for: The mitochondrial RNA polymerase POLRMT promotes skin squamous cell carcinoma cell growth
Source: Cell Death Discov. 2022 Aug 3;8:347. doi: 10.1038/s41420-022-01148-5 (PMC9349297; doi:10.1038/s41420-022-01148-5)

Figure S1. The uncropped blotting images of the study

Figure 1.

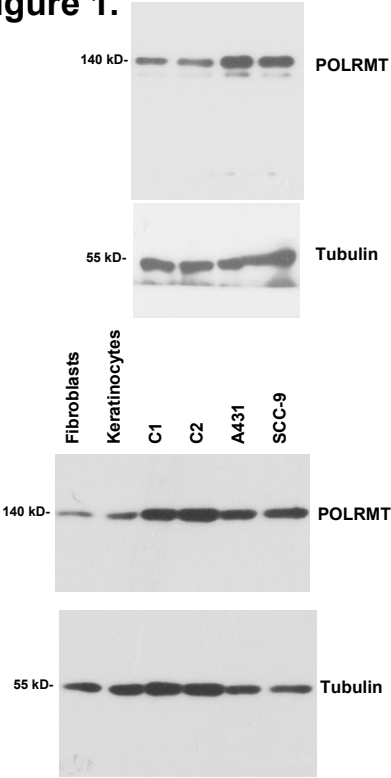

Figure 2.

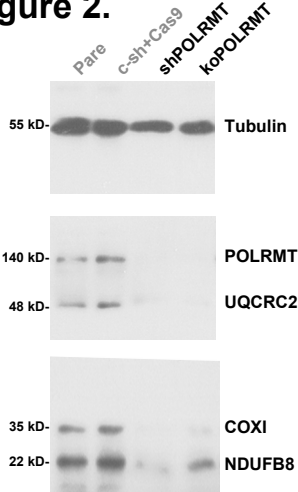

Figure 3.

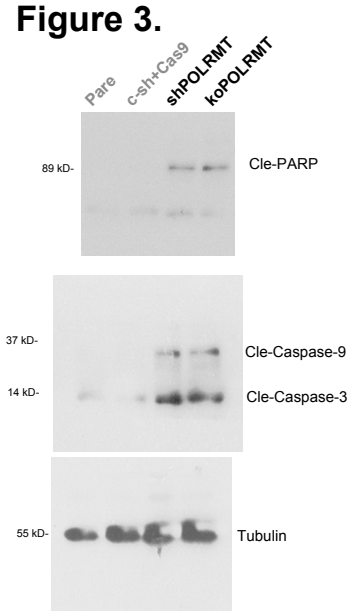

Figure 5.

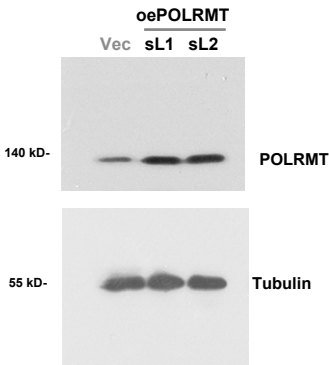

Figure 6.

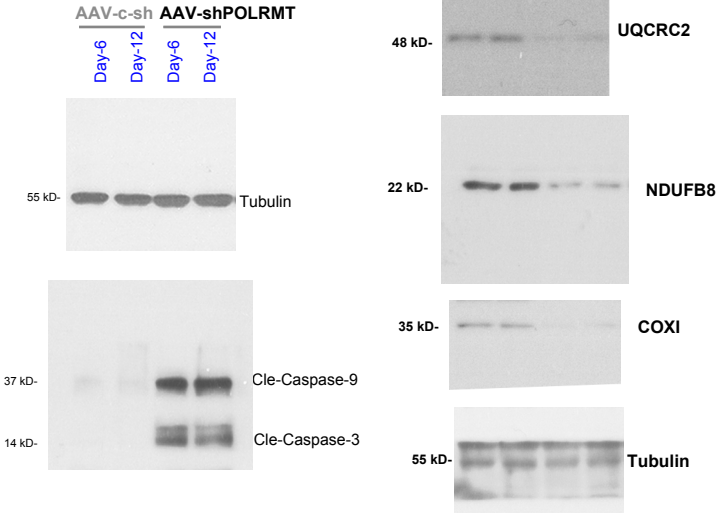

Supplement: Supplementary file 2 — Figure S1 [file 41420_2022_1148_MOESM2_ESM.pdf]
